# The Categorization of Objects With Uniform Texture at Superordinate and Living/Non-living Levels in Infants: An Exploratory Study

**DOI:** 10.3389/fpsyg.2020.02009

**Published:** 2020-08-06

**Authors:** Kosuke Taniguchi, Azumi Tanabe-Ishibashi, Shoji Itakura

**Affiliations:** ^1^Center for Baby Science, Doshisha University, Kyoto, Japan; ^2^Institute of Development, Aging and Cancer, Tohoku University, Miyagi, Japan

**Keywords:** object categorization, superordinate-level categories, living/non-living level categories, category hierarchy, infants

## Abstract

Human infants can categorize objects at various category levels (e.g., as a dog, animal, or living thing). It is crucial to understand how infants learn about the relationships between objects. This study investigated whether 4- to 11-month-old infants can categorize modeled objects at superordinate and living/non-living levels. In this experiment, we presented modeled objects with a uniform texture constructed by a 3D printer in animal, vegetable/fruit, vehicle, and tool categories and measured the time taken to examine novel categories. We investigated infants’ categorization abilities using familiarization/novelty-preference tasks and their pre-linguistic development based on information from their parents. The analyses examined whether infants dedicated more examination time to objects in the new category at superordinate and living/non-living levels for each month of age. The results revealed that the examination time among 4- and 5-month-olds was at chance levels for both superordinate and living/non-living levels, while at 7 months, they showed high preference for the novel category at both category levels. For the superordinate level, the strength of response to living objects increased with linguistic development, while the strength of response to non-living objects did not depend on linguistic development. This indicates that the superordinate-level categorization of living objects depends on both perceptual information and linguistic ability. For the living/non-living level, the examination time for non-living objects increased with linguistic development. This implies that the recognition of non-living objects may depend on the development of object knowledge. The current study suggests that infants can recognize categories at an abstract level before the acquisition of linguistic representations while the category levels that infants can categorize objects are different for living/non-living objects. This may imply that infants learn the concepts of living/non-living via different mechanisms.

## Introduction

We can, at a glance, recognize and categorize an object accurately and quickly (Thorpe et al., 1996). In fact, visual object recognition, which is largely associated with visual processing (Marr, 1982), constitutes a fundamental function of our daily lives. Moreover, one of the main goals of object recognition is object categorization, which depends on the abstraction levels of categories. Rosch et al. (1976) showed that objects at the basic level of the category (i.e., car) were categorized faster than at the superordinate (i.e., vehicle) and subordinate (i.e., Beetle) levels. Rosch et al. (1976) also found that children over 3 years of age were likely to learn object words at the basic level earlier than at other levels. They suggested that children learn object concepts at the basic level first, before subsequently acquiring the different category levels (i.e., the superordinate and subordinate levels).

However, some studies have revealed advantages of categorization at global levels over the basic level category for adults. Macé et al. (2009) showed that categorization at the superordinate level was faster than that at the basic level, using an ultra-rapid categorization task. They suggested that the visual system accesses object representation from a coarse/abstract level to a fine/specific level. The superordinate level categorization can access object representation from early visual information, and therefore permit faster decisions than for basic-level categorizations at short stimulus durations, although superordinate-level categorizations were slower at longer stimulus durations. This advantage of superordinate-level categorization is supported by Taniguchi et al. (2020), who showed that superordinate-level categorization does not depend on information representative of the category, but rather on perceptual information (e.g., complexity of shape). Greater accessibility of superordinate-level categorization has also been suggested by computational theory. Rogers and Patterson (2007) examined categorization performance at three category levels, namely basic, general (i.e., superordinate), and specific (i.e., subordinate), with four deadline conditions in a reaction-time assessment paradigm. They showed that behavior matched the predictions of parallel distributed processing (PDP) theory, indicating that semantic representation at the superordinate-level activates earlier than basic-level categorization, especially with shorter response deadlines (i.e., a requirement for faster responses). Thus, superordinate-level categorization is unlikely to depend on object representation, unlike basic and subordinate levels.

Mandler and McDonough (1993) investigated whether 7- to 11-month-old infants could categorize objects at the basic and global levels. The results showed that at 7 to 11 months, infants could categorize vehicle objects at the basic level (i.e., car from airplane and motorcycle), while they could not distinguish animal objects (i.e., dog from fish and rabbit). At the global level, infants could categorize animals and vehicles regardless of similarities among the categories such as shape and texture. Similarly, Behl-chadha (1996) investigated the ability of 3- and 4-month-old infants in categorizing natural and artifactual objects, and the results indicated that they could categorize artifactual objects at both the basic and the global levels (i.e., furniture vs. animals). Likewise, Quinn and Johnson (2000) examined whether 2-month-olds can categorize objects at the basic (e.g., cat vs. elephant) and global (e.g., mammal vs. furniture) levels using a familiarization/novelty-preference task. Their results indicated that 2-month-olds could categorize at the global level, but not at the basic level. Therefore, infants may acquire global-level categorization earlier than basic-level categorization. This might be because infants’ object categorization at the global level is based not only on perceptual information but on conceptual representation as well. Is global-level categorization influenced by linguistic development? To answer this question, in this study, we investigated whether the recognition of global-level categorization was influenced by linguistic development.

To understand the function of object categorization, it is essential to consider the relationship between the development of object categorization and that of linguistic ability. While some previous studies showed that presenting word labels facilitated categorization learning in infants (Balaban and Waxman, 1997; Fulkerson and Waxman, 2007). other studies indicated that infants’ cognitive development acquires perceptual categorization, which helps acquisition of language accordingly (Mandler, 2004). Therefore, whether infants before the acquisition of language can categorize objects at global levels, such as superordinate and living/non-living levels, is a controversial point. To specify the relationship between linguistic development and object categorization at global levels, this study investigated infants’ response to novel objects at superordinate and living/non-living levels.

Moreover, to understand the properties of the human visual system pertaining to object categorization, it is important to consider categorization at the living/non-living level. The ability to perceive and categorize an object as living is essential, such as when detecting a predator or finding food. Therefore, high sensitivity to living objects might be a characteristic of the human visual system (VanRullen and Thorpe, 2001). Moreover, when studying the category levels of an object, the superordinate-level category is usually defined as an animal, vegetable/fruit, vehicle, and furniture, according to the hierarchy of object category, whereas the more abstract level category can be defined as the living/non-living level. Studies on object categorization have often investigated the processing mechanisms of living/non-living categorization (e.g., Praß et al., 2013). For adults, categorization of items as living/non-living differed between living and non-living objects in behavioral and neuropsychological studies (McMullen and Purdy, 2006; Riddoch et al., 2008; Mahon et al., 2009). For the categorization of living objects, perceptual information is more crucial, whereas contextual and functional information is more critical for non-living objects (Garrard et al., 2001; Cree and McRae, 2003).

The sensitivity to living objects will be high even in infants, if categorizing an object as living is critical for survival. Pauen (2002) investigated whether 10- and 11-month-old infants could categorize living and non-living objects based on their perceived similarity by using an object-examination task with three-dimensional objects. The results indicated that infants were able to categorize living and non-living objects regardless of the similarity between the objects. Therefore, Pauen (2002) proposed that the categorization of living and non-living objects is based on knowledge-based processing. Some researchers have indicated that the structure of object concepts in infants and young children might differ from those of adults. For example, children aged between 3 and 5 years attribute life to non-living things, such as clouds and watches (“animistic;” Piaget, 1929). Herrmann et al. (2012) suggested that this error causes young children’s lower accessibility to biological knowledge, although they already have it. Therefore, the acquisition of living/non-living concepts is predicted to relate with linguistic development than acquisition of the basic- and superordinate-level categories. However, Graham and Poulin-Dubois (1999) demonstrated that infants at the beginning of their linguistic development (approximately one and a half year-old) can learn categorical labels of objects by relying on shapes rather than textures (e.g., colors). They also showed that infants can categorize unknown objects as either animate or inanimate. Considering these findings, it is possible that infants learn categories based on shapes of objects before language acquisition (before 1-year-old). Furthermore, infants might be able to categorize objects at the living/non-living and superordinate levels based on their shapes. Thus, the question of how infants categorize at the living and non-living level is a highly controversial issue. Here, we examined these issues using 3D objects that had various shapes but a unitary texture.

Thus, to determine how the hierarchical structure of object categories is constructed in infants, we exploratively investigated whether infants’ categorization ability is affected by linguistic development and whether objects are living vs. non-living, at both superordinate and living/non-living levels. Accordingly, we conducted familiarization/novelty-preference tasks using the object examination method among 4- to 11-month-olds, which is based on the logic by which infants’ responses during the experiment should vary by the degree of perceptual differences among the stimuli (Pauen, 2002). We investigated differences in response strength by the category level of objects, by presenting a novel object in a different superordinate-level category or in the living/non-living category. Generally, the object examination method is believed to be appropriate for infants aged over 7 months (Mareschal and Quinn, 2001) but we applied this method for comparison with infants younger than 7 months. However, the object examination task used in this study was similar to that in a previous study that involved infants younger than 7 months; we used the familiarization/novelty-preference task (e.g., Quinn et al., 1993). In general, infants show more interest in 3D objects than in static images (Mandler, 2000; Perry, 2015). We, therefore, used the object examination method to examine whether infants can categorize objects at both the superordinate and living/non-living levels using perceptual information, such as that available from 3D shapes with a uniform texture. This study specifies the characteristics of global level categorization in infants, such as superordinate and living/non-living levels, assuming that infants’ categorization is characterized by linguistic development. If infants’ categorization at the global level depends only on perceptual information, then infants’ preference for novel objects will not change with language development. Moreover, if the effects of linguistic development affect responsiveness to living objects, infants’ preference for living/non-living objects will be different.

This study focused on the relation of shape information with category representation to investigate whether categorization at superordinate and living/non-living levels depends on linguistic development and whether objects are living/non-living. Shape information is the most important visual property in object recognition (Marr, 1982; Biederman, 1987; Ullman, 1996). This also applies to infants (Van de Walle et al., 1997). 3D printers can easily create objects with controlled shapes and textures. Therefore, in this study we assessed whether categorization processing from shape information among 4- to 11-month-olds differs between superordinate and living/non-living levels. This study used shape stimuli with a uniform texture produced by 3D printer.

## Materials and Methods

### Participants

Thirty-four Japanese infants from 4 to 11 months old participated in this study (16 males and 18 females; *M* = 228.2 days, *SD* = 66.92). Among them, we will investigate the effect of pre-linguistic development extensively. All participants were recruited from Doshisha University’s waiting list. The design and purpose of the study were explained to the infants’ parents and written informed consent documents were obtained from them before starting the experiment. The ethical community of Doshisha University approved of this research (approval number: 16091).

We calculated the sample size required to test the linear mixed model by using lmmpower function for longpower package in *R*, with 0.80 for beta (power of test) and 0.05 for alpha (significant level). The results indicated that 74 samples were needed. Approximately 19 participants were needed for this experiment, since four trials were repeated for each participant (74/4 = 18.5).

### Stimuli

**FIGURE 1:**
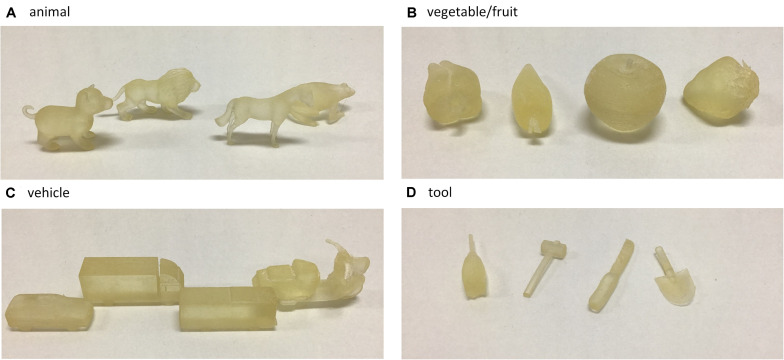
The stimuli used in this experiment. The objects used in the animal **(A)**, vegetable/fruit **(B)**, vehicle **(C)**, and tool **(D)** categories.

The stimulus set was selected from animal, vegetable/fruit, vehicle, and tool categories which consisted of modeled objects of living (i.e., animal and vegetable/fruit; living object) and non-living (i.e., vehicle and tool; non-living object). A total of 16 modeled objects were presented in the experiment (animal: dog, lion, horse, and frog; vegetable/fruit: apple, strawberry, green pepper, and carrot; vehicle: car, bus, truck, and motor scooter; tool: hammer, saw, scoop, and broom; Figure 1). For animal categories, the objects were animals standing on their four legs, which represented complex shapes. The vegetable/fruit objects consisted of simple shape, such as rectangles or spheres. The vehicle objects involved rectangular shapes with large volume. The tool category consisted of objects that were essentially small-volume cylinders. The stimuli were constructed by a 3D printer (Stratasys, Objet30 Prime). Each stimulus size was set to approximately 8–15 cm on one side at maximum. The modeled objects were made of acrylic, translucent resin.

### Procedure

Infants were seated on their parents’ lap in front of the table. The experimenter was seated on the floor at the opposite side of table. The experimenter presented a stimulus (for familiarization blocks) or two stimuli (for test blocks) within the infants’ reach. If an object fell off the table, the experimenter picked it up and put it back on the table as soon as possible. The task comprised four familiarization and four test blocks. These blocks were alternated (i.e., first familiarization block, then first test block, second familiarization block, …fourth test block). When one block was finished, the next block was started immediately. The experiment was paused if the infant cried. Before starting the experiment, short warm-up trials were conducted by presenting blocks of various geometric shapes (i.e., cylinder, triangle pole, and square pole). The experimenter did not label any of the objects, and the parents were asked not to interact with the infants during the session. Completion of all blocks required approximately 30 min.

For familiarization blocks, one of the three stimuli in a category was presented randomly. The infants could explore the object any way they wished (e.g., play, explore, and ignore). The stimulus was presented for a minimum of 20 s, and if the infants showed interest in it after 20 s, they could continue their examination until 180 s. Then, the next stimulus was presented. Three stimuli in a category were presented once or twice. If infants did not respond to a new object, the familiarization block was terminated. In total, three to six trials were conducted in a familiarization block.

Test blocks were conducted immediately after a familiarization block was completed. For test blocks, two stimuli were presented simultaneously beside each other: one was the novel object in the category presented in the familiarization block and the other was an object from a novel category. We randomly assigned the positions of the two stimuli. The novel category was chosen based on superordinate (e.g., animal vs. vegetable/fruit and vehicle vs. tool) or living/non-living levels (e.g., animal vs. vehicle and vegetable/fruit vs. tool). The infants could explore the objects the same way as in the familiarization blocks. A test block consisted of one trial. After the trial was finished, the next familiarization block was started.

The behavior of infants in the experiment was recorded using a video camera placed in front of them. After four test blocks were completed, the experimenter orally asked infants’ parents questions on the pre-linguistic scale and recoded their answers.

### Pre-linguistic Scales

To investigate the effects of linguistic development, we asked parents for their responses to the Nagao Pre-Linguistic Scales (Nagao et al., 1990). The scales were used in 12 questions selected from the stage of language development and representing the function of language at 1–12 months, which involved development of vocal expressions and symbolistic functions. Vocal expressions also involved infants’ communication ability through aspects such as emotion expression and attracting mother’s attention (but not by crying). Nagao et al. (1990) argued that these communication abilities indicate the differentiation of objects and are associated with the development of conceptual function. The score on the pre-linguistic scales, therefore, indicated the degree of development in conceptual function.

The score rating was followed by Nagao et al. (1990): If it certainly acknowledged, the score was 1. If it had started appearance but it does not completely, the score was 0.5. If it had not appeared yet, the score was 0.

### Video Coding

One coder for the familiarization blocks and two coders for test blocks measured the examination time for each trial. They were not aware of the purpose of this study. For familiarization blocks, the coder recorded the examination time, defined as the time during which an infant examines an object by using observation and touch. Examination time was measured from the beginning of the stimulus presentation until the infant stopped examining the stimulus. For test blocks, the coders recorded the examination time of each stimulus over 20 s from the time of first response to a stimulus, such as the first look or touch. The examination time may indicate the strength of response to the stimulus (Pauen, 2002).

### Statistical Analysis Methods

We conducted repeated-measures linear mixed-models using the lme4 (Bates et al., 2015) and lmerTest (Kuznetsova et al., 2017). Packages in R to compare the difference between group means in repeated measure and mixed design. This procedure produces results similar to those of ANOVA and *t*-tests. In a linear mixed-model, the random participant effect is constructed using the correlations between the observations of the participants. Linear mixed-models are generally robust against missing values (Baayen et al., 2008).

## Results

Data from two female infants (an 8-month-old and an 11-month-old) were excluded from the analyses, because they did not show any response to the stimuli. The mean examination time in trials between coders for test blocks provided the raw data for further analyses because intercoder reliability was high (*r* = 0.94, range: 0.93–0.96).

### Familiarization Trials

**FIGURE 2:**
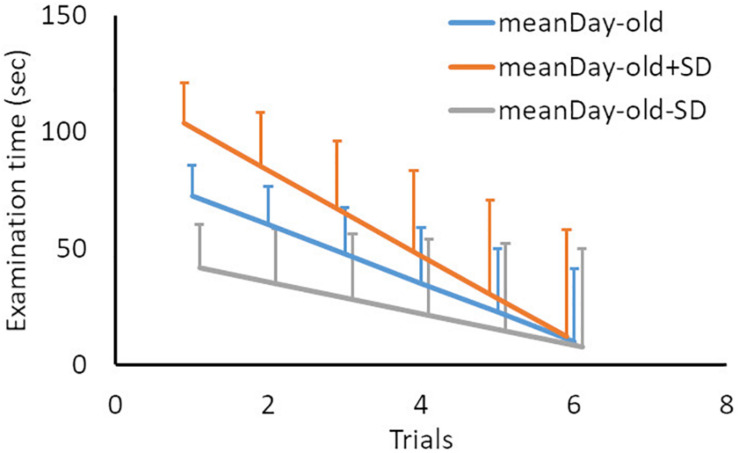
Mean examination time and the prediction from the linear mixed-model in the familiarization test, as a function of familiarization trial. “meanDay-old” indicates the mean age of participants in days (221.57), while “meanDay-old ± *SD*” denotes the mean age ± 1 *SD* (67.15), to show the interaction effect between age in days and trial number. Error bars indicate standard errors.

To examine whether infants were habituated to the presented stimulus or category, the model set examination time as the dependent variable, trials in a block (centered at first trial: First trials) as the continuous variable, days-old [centered at the mean (227.45 days) of days-old: days-old] as the fixed variable, and infant ID as the random variable. The analyses involved 475 observations from 32 participants. The analysis revealed a significant effect of First trials and Days-old [First trials: beta = -12.53, *t*(453) = -6.67, *p* < 0.001; Days-old: beta = 0.46, *t*(44) = 4.73, *p* < 0.001] and the interaction between them [beta = -0.09, *t*(455) = -3.08, *p* < 0.01], indicating that examination time decreased with trials number (Figure 2 and Table 1).

**TABLE 1:** The estimations of the linear mixed-model based on the familiarization trials.

	Beta	*SE*	*df*	*t*-value	*p*
Intercept	72.54	6.60	45.14	11.00	<0.001
Days-old	0.46	0.10	44.75	4.73	<0.001
First trials	–12.53	1.88	453.79	–6.67	<0.001
Days-old × first trials	–0.09	0.03	455.90	–3.08	0.002

### Test Trials

The analyses involved 119 observations from 32 participants. As preliminary analysis, we conducted a hierarchical multiple regression analysis of the linear mixed model in order to investigate whether there are differences in infant preferences by category of novel object. In the model, preference scores were used as the dependent variable, while the category of novel object was used as a fixed variable and the infant’s ID was used as a random variable. The results showed that the main effect of the category was not significant [χ ^2^ (3) = 5.30, *n.s.*].

**FIGURE 3:**
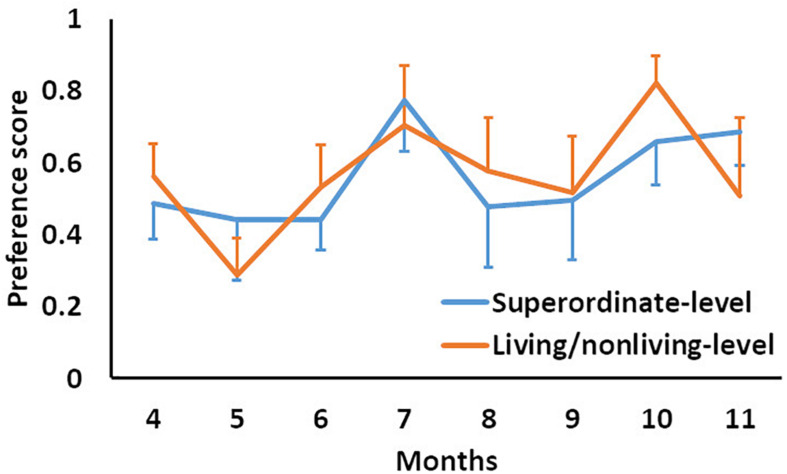
Mean preference score as a function of months and category level. Error bars indicate standard errors.

The analysis focused on the intercept because we examined whether or not the preference score was more than the chance-level (i.e., 0.5). We set the months-old variable as dummy variable centered at each month, to investigate effects of age change. The series of analyses revealed that the effect of the intercept was significant in 7-month-olds at both superordinate and living/non-living levels [beta > 0.22, *t*(110) > 2.58, *p* < 0.05]. The intercept effect in 10-month-olds was significant for the superordinate-level [beta = 0.25, *t*(110) = 2.00, *p* < 0.05], while the intercept effect approached significance in 10-month-olds for the living/non-living level [beta = 0.23, *t*(110) = 1.88, *p* < 0.1]. The intercept effect was not significant in infants of other ages. In the contrast, the effect of category level was not significant in infants of any age (Figure 3).

**FIGURE 4:**
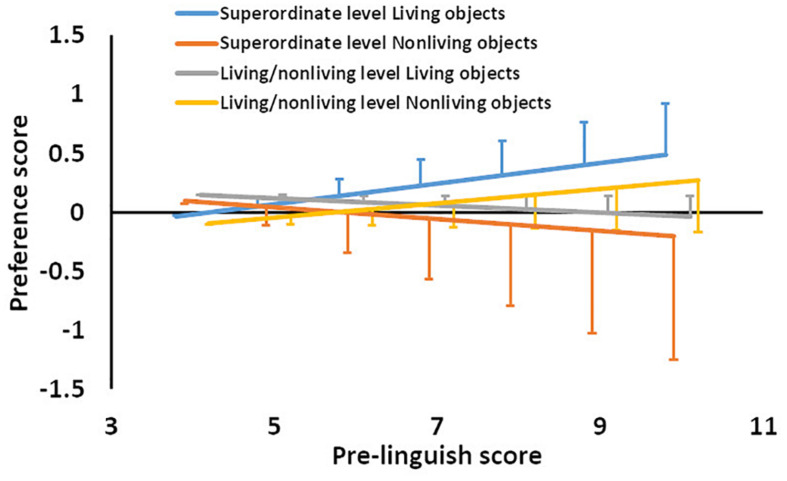
The prediction from the linear mixed-model as a function of pre-linguistic score, category level, and living/non-living object. Error bars indicate standard errors.

To test the difference between the categorization of superordinate and living/non-living levels by linguistic development and object properties in the living/non-living category for a new category, a series of a linear mixed modeling effects was conducted. The model set the preference score (centered at 0.5) as the dependent variable. Pre-linguistic score (centered at mean: linguistics) was set as the continuous variable, categorization level (superordinate vs. living/non-living level: category level) was set as the fixed variable, living/non-living category of objects (living object vs. non-living object: object category) were set as dummy variables, and the interactions among them were also included in the model. Infant ID was set as a random effect. The analyses involved 119 observations from 32 participants. Note that the effect of age in days-old was omitted from the fixed variables in these analyses, to avoid multi-collinearity with pre-linguistic score [*r*(117) = 0.67, *p* < 0.001]^1^. The results revealed significant two-way interactions between pre-linguistic score and category level [beta = 0.12, *t*(111) = 2.77, *p* < 0.01] and between pre-linguistic score and object category [beta = 0.09, *t*(111) = 2.09, *p* < 0.05]. The three-way interaction among pre-linguistic score, category level, and object category was also significant [beta = -0.23, *t*(111) = -3.76, *p* < 0.001; as shown in Table 2]. Figure 4 shows the results of analyses of simple main effects of the three-way interaction, showing that preference scores for living objects at the superordinate level were higher than those at the living/non-living level for infants with higher linguistic ability. In addition, preference scores for living objects were higher than those for non-living objects at the superordinate level of categorization for infants with higher linguistic ability. As Figure 4 shows, the linguistic effects were revealed at the superordinate-level categorization of living objects and at the living/non-living level of non-living objects.

**TABLE 2:** The estimations of the linear mixed-model based on the test trials.

	Beta	*SE*	*df*	*t*-value	*p*
Intercept	0.08	0.06	111.00	1.32	0.191
Pre-linguistic score	–0.03	0.03	111.00	–1.01	0.317
Superordinate-level	0.07	0.09	111.00	0.80	0.424
Non-living object	–0.05	0.09	111.00	–0.55	0.585
Pre-linguistic score × superordinate level	0.12	0.04	111.00	2.77	0.007
Pre-linguistic score × non-living object	0.09	0.04	111.00	2.09	0.039
Superordinate level × non-living object	–0.11	0.12	111.00	–0.92	0.359
Pre-linguistic score × superordinate level × non-living object	–0.23	0.06	111.00	–3.76	< 0.001

## Discussion

In this study, we investigated whether categorization at both the superordinate and living/non-living levels depends on linguistic development and whether objects are living/non-living in 4- to 11-month-old infants. The results of this study indicated that infants older than 7 months could categorize objects at both the superordinate and living/non-living levels. However, the strength of response was associated with an interaction between living/non-living objects and category levels. Infants with higher pre-linguistic scores showed higher preference scores for living objects at the superordinate level than at the living/non-living level of categorization. Infants at 7 months could distinguish the category at both the superordinate and living/non-living levels, although their processing was similar. The processing of superordinate and living/non-living levels may differ owing to linguistic development.

For superordinate-level categorization, the effects of linguistic development differed between living and non-living objects: the linguistic score did not influence preference scores for non-living objects, although it did increase the examination time for living objects. This might indicate that superordinate-level categorization depends on both perceptual information and linguistic ability for living objects. These results are consistent with a previous study that showed both pre-existing representation and perceptual information related to infants’ categorization abilities (Smith, 2000; Pauen, 2002). Therefore, infants categorize living objects at the superordinate-level depending not only on perceptual information but also on object concepts.

For non-living objects at the superordinate-level categorization, no effect of linguistic ability was found. This might be because perceptual shape information plays an important role in the categorization of non-living objects. Some studies found that 2-year-old children categorize non-living objects based on functional features (e.g., Deborah et al., 2004; Casler and Kelemen, 2007), which indicates that functional features are important for shaping the concepts of non-living objects. The current study also indicated that 7-month-olds can categorize objects at both superordinate levels. Thus, it might be possible for infants to perceive functional features from the shape of objects at circa 7 months of age.

For categorization at living/non-living level, preference scores for non-living objects increased correspondingly to pre-linguistic scores, while those for living objects were unaffected by pre-linguistic scores. These results suggest that perceptual information is more effective for categorization of living objects at the living/non-living level, because the shape components of living objects were more complex than those of non-living objects. That is, the living category consisted of rounded and curved surfaces, while the non-living category consisted of straight lines, right angles, and corners. However, the response strength did not different according to linguistic development for non-living objects. Infants generally prefer more complex, informative objects over other types of objects (e.g., Fantz, 1958). Thus, response strength is expected to be greater for living objects. Moreover, 10-month-olds cannot categorize objects at the living/non-living level but can at the superordinate level. Therefore, more developed object knowledge than that exhibited by 11-month-olds might be necessary for infants to categorize at the living/non-living level.

In this study, we used familiarization/novel-preference tasks with 3D objects to measure the strength of category preference by considering both fixation and the touching of objects (Oakes et al., 1991; Oakes and Tellinghuisen, 1994). The results indicated that the 4- and 5-month-old infants did not show any preference for the novel category, which was inconsistent with Quinn and Johnson (2000). Mareschal and Quinn (2001) argued that young infants form an exclusive category when the examined objects have low variability in perceptual features. As we used objects with a uniform texture in the current study, young infants might not have formed inclusive categories of several objects. Moreover, the familiarization trials showed that 4- and 5-month-olds also did not show habituation to objects, because they showed weak responses in both familiarization and test trials in this study. Therefore, for 4- and 5-month-old infants, shape information might not be sufficient for categorization.

In the current study, the recognition of living objects was found at the living/non-living level of categorization. In some studies, recognition of living objects was not found. For example, VanRullen and Thorpe (2001) investigated whether living objects (i.e., mammals) were categorized faster than non-living objects (i.e., means of transport), using ultra-rapid categorization tasks. They revealed that response speed for living objects was not greater than that for non-living objects. This might have been due to a category-level effect as, in the current study, we found different effects of living objects between superordinate and living/non-living levels. Further studies will be needed to clarify the living objects effect.

Additional issues of categorization at a global level relate to the variety of objects within a category. For example, the shapes of birds and fishes are very different from the shapes of the animals used in this study. In this study, the stimuli of objects were limited, and infants were familiarized with a single category in a familiarization block. Therefore, the question remains as to how infants categorize these different shape objects within the same category. However, the current study indicated that categorization by infants might depend not only on shape information, but also on linguistic development of objects. These findings provide insight into the how the hierarchical structure of object concepts is acquired.

Recognizing objects is a critical life skill (e.g., for finding food, grasping a bottle, or communicating with others). Some diseases could cause deficits in object recognition ability. For example, patients with semantic dementia show gradually increasing deficits in conceptual knowledge regarding word and object meanings. Such patients sometimes show impaired categorization at basic and subordinate levels, but intact categorization at the superordinate level (Rogers and Patterson, 2007). Furthermore, individuals with autism spectrum disorder (ASD) show difficulties in recognizing atypical objects (Gastgeb and Strauss, 2012). This may lead to problems for individuals with ASD in solving problems that involve abstract content (Alderson-Day and McGonigle-Chalmers, 2011). According to these studies, the deterioration of object recognition may cause difficulties with daily living. The current study implies that the learning of object concepts may differ between living and non-living objects, given the different response strength at the category level. Difficulties in daily living might be caused by distortion of the structure of object concepts. Thus, the current study contributes to extant work by revealing correlations between object recognition ability and other abilities, such as grasping a bottle, pointing an object, and communicating with others.

To summarize, this study investigated how 4- to 11-month-old infants categorized objects at the superordinate and living/non-living levels, using 3D objects with a uniform texture. We conducted object examination trials and measured the duration of infants’ examination of the stimuli. The results showed that preference of a novel category was influenced by whether objects were living/non-living and linguistic development. For the superordinate-level category, the strength of response to living objects increased with linguistic development, but that to non-living objects was unaffected by linguistic development. This indicates that the superordinate-level categorization of living objects depends on both perceptual information and linguistic ability, consistent with the idea of superordinate-level recognition. For the living/non-living category, preference scores did not differ between living and non-living objects. This implies that categorization at the living/non-living level may depend on more developed object knowledge and finer perceptual discrimination. The current study suggests that infants can recognize categories at an abstract level, using not only shape information of objects, but also linguistic representations.

## Data Availability Statement

The datasets generated for this study are available on request to the corresponding author.

## Ethics Statement

The studies involving human participants were reviewed and approved by the ethical community of Doshisha University. Written informed consent to participate in this study was provided by the participants’ legal guardian/next of kin.

## Author Contributions

KT and AT-I designed this study and wrote the original draft. KT conducted the experiments and analyzed the results. SI supervised this project. All authors contributed to revisions, read, and approved the submit version.

## Conflict of Interest

The authors declare that the research was conducted in the absence of any commercial or financial relationships that could be construed as a potential conflict of interest.

## Footnotes

1 We did not analyze the data by dividing the infants into younger and older categories because we aimed to investigate the effects of pre-linguistic development exploratively. Moreover, we also conducted the repeated measure mixed model analysis by using a similar procedure in the analysis of test trials, except that the age group [younger than 7 months (i.e., 4- to 6-month-olds) vs. 7 months or older (i.e., 7- to 11-month-olds)] rather than pre-linguistic score was set as a dummy dependent variable. The findings indicated results almost identical to those indicated in Figure 3 and Table 2 (see Supplementary Figure 1 and Supplementary Table 1).
