# Supplementary material for: The Categorization of Objects With Uniform Texture at Superordinate and Living/Non-living Levels in Infants: An Exploratory Study
Source: Front Psychol. 2020 Aug 6;11:2009. doi: 10.3389/fpsyg.2020.02009 (PMC7424027; doi:10.3389/fpsyg.2020.02009)
Supplement: Supplementary file 1 [file Table_1.pdf]

Supplementary Material

1 Supplementary Figures and Tables

1.2 Supplementary Tables

Supplementary Table 1| The estimations of the linear mixed-model based on supplemental analysis.

	Beta	*SE*	*df*	*t*-value	*p*
Intercept	0.08	0.08	111.00	0.99	0.33
Before 7-month-old	-0.00	0.13	111.00	0.00	1.00
Superordinate-level	0.19	0.11	111.00	1.71	0.09
non-living object	0.05	0.11	111.00	0.40	0.69
Before 7-month-old × Superordinate-level	-0.31	0.18	111.00	-1.73	0.09
Before 7-month-old × non-living object	-0.23	0.18	111.00	-1.27	0.21
Superordinate-level × non-living object	-0.34	0.16	111.00	-2.21	0.03
Before 7-month-old × Superordinate-level × non-living object	0.62	0.25	111.00	2.46	0.02
