# Supplementary material for: The Categorization of Objects With Uniform Texture at Superordinate and Living/Non-living Levels in Infants: An Exploratory Study
Source: Front Psychol. 2020 Aug 6;11:2009. doi: 10.3389/fpsyg.2020.02009 (PMC7424027; doi:10.3389/fpsyg.2020.02009)
Supplement: Supplementary file 2 [file Image_1.pdf]

Supplementary Material

1 Supplementary Figures and Tables

1.1 Supplementary Figures

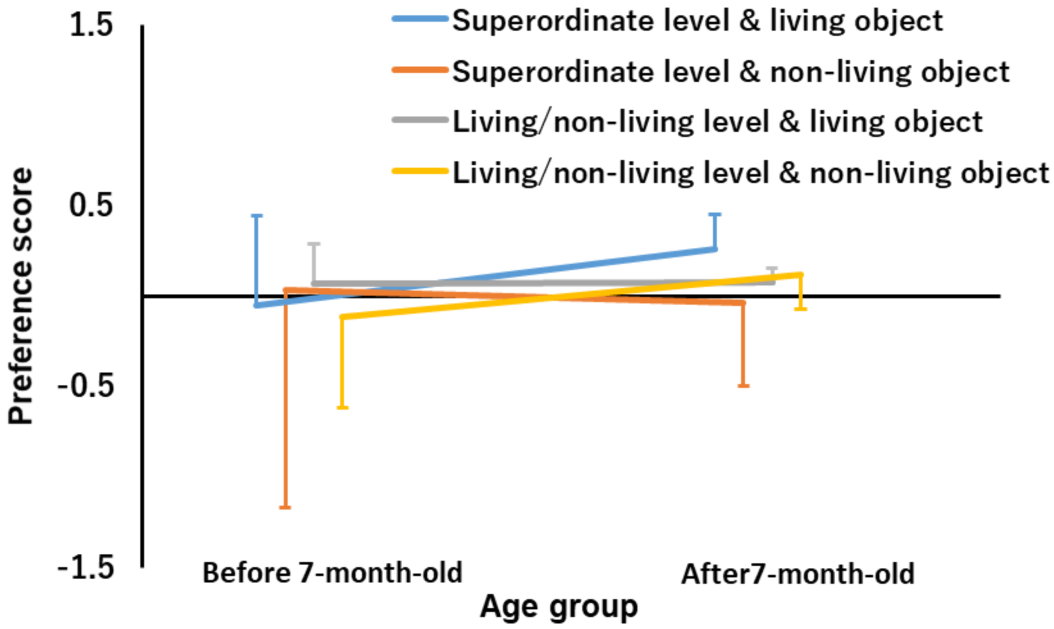

Supplementary Figure 1. The prediction from the linear mixed-model as a function of age group, category level, and living/nonliving object. Error bars indicate standard errors.
